# Bath Breakfast Project (BBP) - Examining the role of extended daily fasting in human energy balance and associated health outcomes: Study protocol for a randomised controlled trial [ISRCTN31521726]

**DOI:** 10.1186/1745-6215-12-172

**Published:** 2011-07-08

**Authors:** James A Betts, Dylan Thompson, Judith D Richardson, Enhad A Chowdhury, Matthew Jeans, Geoffrey D Holman, Kostas Tsintzas

**Affiliations:** 1 Human Physiology Research Group, Department for Health, University of Bath, BA2 7AY, UK; 2 Department of Biology and Biochemistry, University of Bath, BA2 7AY, UK; 3 School of Biomedical Sciences, Queen's Medical Centre, Nottingham, NG7 2UH, UK

## Abstract

**Background:**

Current guidance regarding the role of daily breakfast in human health is largely grounded in cross-sectional observations. However, the causal nature of these relationships has not been fully explored and what limited information is emerging from controlled laboratory-based experiments appears inconsistent with much existing data. Further progress in our understanding therefore requires a direct examination of how daily breakfast impacts human health under free-living conditions.

**Methods/Design:**

The Bath Breakfast Project (BBP) is a randomised controlled trial comparing the effects of daily breakfast consumption relative to extended fasting on energy balance and human health. Approximately 70 men and women will undergo extensive laboratory-based assessments of their acute metabolic responses under fasted and post-prandial conditions, to include: resting metabolic rate, substrate oxidation, dietary-induced thermogenesis and systemic concentrations of key metabolites/hormones. Physiological and psychological indices of appetite will also be monitored both over the first few hours of the day (i.e. whether fed or fasted) and also following a standardised test lunch used to assess voluntary energy intake under controlled conditions. Baseline measurements of participants' anthropometric characteristics (e.g. DEXA) will be recorded prior to intervention, along with an oral glucose tolerance test and acquisition of adipose tissue samples to determine expression of key genes and estimates of tissue-specific insulin action. Participants will then be randomly assigned either to a group prescribed an energy intake of ≥3000 kJ before 1100 each day or a group to extend their overnight fast by abstaining from ingestion of energy-providing nutrients until 1200 each day, with all laboratory-based measurements followed-up 6 weeks later. Free-living assessments of energy intake (via direct weighed food diaries) and energy expenditure (via combined heart-rate/accelerometry) will be made during the first and last week of intervention, with continuous glucose monitors worn both to document chronic glycaemic responses to the intervention and to verify compliance.

**Trial registration:**

Current Controlled Trials ISRCTN31521726.

## Background

It is over four decades since Pavel Fábry and colleagues published their studies showing positive correlations between infrequent daily meal patterns and overweight, hypercholesterolaemia, impaired glucose tolerance and ischaemic heart disease [1]. Since then, it is certainly notable that the increased prevalence of overweight and obesity in Western Societies has been paralleled by a progressive decline in breakfast consumption [2]. However, subsequent scientific literature has not substantially progressed understanding beyond the initial cross-sectional associations published in the late 1960s. While subsequent cross-sectional and/or longitudinal investigations have undoubtedly clarified certain aspects of these correlations and confirmed such relationships across a wider population range, it is impossible to establish any degree of causality from these data due to major confounding variables; for example, the fact that frequent breakfast consumers also tend to exhibit numerous other healthful lifestyle choices such as lower fat/alcohol intakes, higher fibre/micronutrient intakes, not smoking and also a tendency to exercise more [3-11].

In terms of existing cross-sectional evidence linking increased adiposity with infrequent or insufficient breakfast consumption, the wealth of available literature does conform to the stated positive relationship [1,7,11-21]]. However, the majority of these large-scale epidemiological studies have involved self-reported dietary records, which are known to underestimate absolute energy intake to a greater extent for more overweight individuals [22]. Indeed, Summerbell *et al*. (1995) reanalysed the data from 3 existing studies across a range of age groups and found that the commonly reported associations between frequent daily meal patterns/high energy intake at breakfast and overweight do not persist once corrected for 'unreasonably low' energy intakes [23]. These corrected results therefore fall into agreement with the one other existing cross-sectional study which found no relationship between daily meal frequency and percent body fatness as determined by hydrostatic weighing, in which dietary records were managed on an individual basis via telephone [24]. Notwithstanding such methodological issues, it might be suggested that one benefit of a frequent meal pattern would be a lasting satiating effect, possibly related to the high fibre content of many breakfast foods [25]. Indeed, at least three intervention studies are consistent with this reasoning [26-28], although many cross-sectional studies have not observed any difference in overall energy intake between frequent and infrequent breakfast consumers [12,13,17,29] or have even found higher energy intakes associated with breakfast consumption [13,15,16,20]. These latter studies necessarily implicate some interaction between energy intake and energy expenditure (i.e. if breakfast consumers are leaner), although the only evidence for such an effect comes from the study by Wyatt *et al*. (2002), in which self-reported physical activity was higher amongst individuals who reported having breakfast on more than 3 days each week [29]. Ultimately, the balance of currently available cross-sectional evidence cannot provide information regarding the direction or even the existence of any causal relationship between breakfast consumption and weight change. Moreover, issues regarding the accurate measurement of changes in energy intake may even question the validity of these findings.

To gain greater insight into this field, it is necessary to move towards higher levels of evidence beyond simple cross-sectional observations. Fortunately, five of the studies that have documented baseline correlations between weight status and breakfast frequency have also conducted longitudinal examinations of breakfast habits and weight change over between 3 and 13 years in both children [11,18,20] and adults [19,21]. In contrast to the cross-sectional evidence detailed above, a far less consistent pattern of findings is apparent. Barton *et al*. (2005), Timlin *et al*. (2008) and Bazzano *et al*. (2005) have all reported a reduced prospective risk of weight gain with frequent breakfast consumption over 5-10 years follow-up in children [18], adolescents [11] and adults [19], respectively; while Kant and colleagues found no relationship whatsoever between weight change over 8-10 years and daily meal pattern either at baseline or at follow-up, although an infrequent daily meal pattern does not necessarily indicate that breakfast was omitted [21]. Interestingly, in that study the authors also note that daily meal pattern was unrelated to self-reported physical activity either at baseline or at follow-up, although no actual data are reported. The balance of longitudinal evidence in this area becomes yet more equivocal when considering the study by Berkey *et al*. (2003), in which a large cohort of children between the ages of 9 and 14 were monitored annually over three years [20]. Importantly, this study also stratified participants according to weight and actually found omission of breakfast to be positively associated with prospective weight *loss *in overweight children. In contrast, normal weight children in this study displayed a trend in the opposite direction in that more weight was *gained *over time when breakfast was always omitted rather than consumed. It is also noteworthy that recent longitudinal evidence has reported detrimental cardiovascular and metabolic health outcomes in association with breakfast skipping even when adjusted for physical activity status and increased body fatness [i.e. waist circumference; [30]]. Overall, the longitudinal studies summarised here question the causal nature of cross-sectional associations between breakfast and adiposity but also introduce the interesting possibility that the strength and direction of this relationship may be mediated by current weight status.

Evidence from randomised controlled trials in this area comes from a wide range of innovative research designs with varied research questions. Specifically, many of these studies have focused on either consuming a low energy breakfast or reducing the number of meals consumed on a daily basis, as opposed to the complete omission of breakfast *per se *[27,31,31-44]. In terms of the former, low energy breakfasts have been found to increase subsequent energy intake during a test meal 4 hours later [34] but not sufficiently to compensate for the reduced energy intake at breakfast [36,45]. In fact, even if overall energy intake is unaffected, it seems that reducing energy intake at breakfast for 6 weeks may actually result in greater reductions in body fat mass but lower overall weight loss due to the preservation of lean tissue mass [35]. Conversely, those studies which have varied the number of meals consumed on a daily basis from between 1 and 9 meals per day have found no effect on energy intake, resting energy expenditure or change in body mass [27,31-33,46-48], although one recent study has reported an increase in fat mass with omission of an afternoon meal for 28 days [47] and another has detected higher overall energy intake from meals of prescribed content during a 14 day period of breakfast omission [26]. The effects of varied meal frequency on dietary-induced thermogenesis (DIT) are even less consistent, with reports of DIT being either unaffected [27,40,41], increased [37,38] or in fact decreased [32,39] by an infrequent daily feeding pattern. In terms of selected health outcomes typically associated with chronic weight change, causal evidence is also inconsistent with what might be expected based upon cross-sectional observations. For example, there is some tentative evidence that a regular daily meal frequency (6-9 meals per day) for 2 weeks can improve insulin sensitivity and blood lipid profiles relative to more chaotic and/or less frequent feeding patterns [43,44]. It is therefore intriguing that intermittent 24 hour fasting every other day for 2 weeks also has the capacity to improve insulin sensitivity, even without any significant short-term reduction in body mass [49]. However, such alternate-day fasting also reduces resting metabolic rate, so may favour weight gain and associated consequences in the long-term [50].

Nonetheless, as mentioned previously, neither varied meal frequency nor reduced energy intake at breakfast necessarily precludes the ingestion of food in the morning to interrupt an overnight fast. It is therefore very interesting that so few randomised controlled trials have directly contrasted the impact of omitting breakfast (i.e. extending the daily overnight fast) from an otherwise matched diet in terms of components of energy balance and subsequent weight loss [26,46,51]. One of these studies has already been discussed in relation to the observed increase in energy intake when breakfast was omitted from the habitual diet [26] but also reported evidence of impaired glycaemic control and elevated blood lipids with breakfast omission, despite the fact that body mass was unaffected. These findings are consistent with the more recent work of Stote *et al*. (2007) in relation to changes in body mass over 8 weeks of extended morning fasting [46], along with metabolic data taken from the same study [52]. The third trial specific to inclusion/omission of breakfast from the habitual diet also involved prescribed dietary intake but with restricted energy intake (i.e. ~ 5000 kJ·d^-1^) over a period of 12 weeks, provided either as 3 meals each day (including a cereal breakfast) or with 2 meals each day (including an additional bran muffin with each meal to maintain energy/fibre intake) [51].

Importantly, the authors of this latter study also stratified participants according to their usual breakfast eating habits at baseline and found habitual breakfast consumers lost more weight by omitting breakfast while habitual breakfast non-consumers lost more weight by introducing breakfast. This study therefore indicates that the success of a weight loss programme can be mediated to an extent by the degree to which that programme differs from each individual's usual dietary habits but also revealed that not consuming breakfast increases the probability of impulsive snacking throughout the day, which is known to be associated with a poorer overall dietary profile [53]. It should be noted, however, that all three of the studies cited above have involved some degree of prescription or restriction in terms of dietary intake, so may not therefore be sensitive to potential 'real-world' alterations in eating behaviour and, furthermore, only the study by Stote *et al*. (2007) has attempted to quantify any effect of interventions on free-living energy expenditure. In that study, accelerometers (Actigraph™) were used to monitor weekly physical activity counts and it was reported that there was no evidence of any difference in physical activity between treatments [46]. However, this measurement tool has been shown to lack reliability when applied to free-living conditions [54] and may not therefore have been sufficiently sensitive to detect subtle alterations in spontaneous low-to-moderate intensity physical activity [55], which intuitively could be considered as the most responsive element of physical activity to modified eating patterns.

What becomes most clear when reviewing the above literature is that very few existing studies have actually attempted to quantify the impact of breakfast on the most malleable component of energy expenditure, namely physical activity energy expenditure [27,29,31,38,42,46]. Even amongst these six studies, it is notable that one simply reported cross-sectional associations based upon a self-administered physical activity questionnaire [29], while four others were randomised controlled trials that directly assessed energy expenditure using a whole-body calorimeter [although this method understandably restricts participants' spontaneous free-living physical activity; [27,31,38,42]]. Therefore, aside from the aforementioned study by Stote *et al*. in which overall physical activity level was estimated using accelerometers [46], only Verboeket-van de Venne *et al*. (1993) have provided data regarding changes in daily physical activities with varied feeding frequency (i.e. 2 versus 7 meals·d^-1^). Physical activity levels in that study were determined as the difference between resting energy expenditure measured using the whole-body calorimeter and average daily free-living energy expenditure measured using the doubly-labelled water technique, which revealed no effect of feeding frequency [38].

A central aim of this project is therefore to conduct a trial in accordance with current CONSORT guidelines which will employ contemporary measurement tools (i.e. combined heart rate and accelerometry) to generate objective and reliable data regarding physical activity energy expenditure under free-living conditions in response to varied daily meal frequency (i.e. daily breakfast versus extended fasting). In contrast to doubly-labelled water, this approach is not limited only to monitoring total energy expenditure but also permits specific evaluation of physical activity profiles in terms of the precise intensity and duration of various daily activities. This novel aspect of the work is complemented by both laboratory- and field-based assessments of all other components of energy balance, along with integration of various other techniques not previously combined in relation to this dietary intervention. In particular, changes in body composition will be accurately monitored via repeated DEXA scanning, continuous glucose monitors will provide a highly sensitive and meaningful analysis of glycaemic control with modified feeding patterns and, lastly, detailed blood/adipose tissue analyses will provide the most mechanistic information to date on this topic, thus establishing the existence and direction of any causal relationships between variables. In summary, there is some support for a positive relationship between extended daily fasting and energy balance, which can lead to a range of inter-related negative health consequences, and this project aims to address the casual nature of these associations via the following objectives:

*i.) To examine the impact of a single extended fast on the acute metabolic and behavioural mechanisms of short-term energy balance regulation.*

*ii.) To determine whether habitual extended fasting is causally related to chronic positive energy balance and establish the mechanisms through which this relationship may operate.*

*iii.) To translate the effects of habitual extended fasting into selected health outcomes related to chronic positive energy balance (i.e. adiposity, insulin resistance and cardiovascular disease).*

## Methods/Design

### Approach to the Research Question

**Figure 1:**
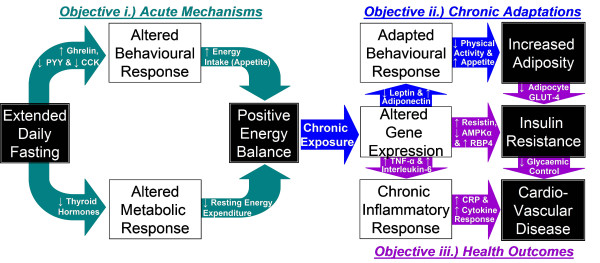
**Proposed mechanistic pathway underlying the relationships between extended daily fasting, energy balance and health outcomes**.

As illustrated in Figure 1, extended daily fasting has the potential to impact upon adiposity, insulin resistance and cardiovascular disease via the acute influences of a single extended daily fast on a given day in terms of contributing to a more positive daily energy balance (***Objective i***) but also via the chronic metabolic and/or behavioural adaptations which may occur with habitual exposure to extended daily fasting over a more prolonged period (***Objective ii***). This project will take advantage of recent technological advances to comprehensively assess all aspects of energy balance and the physiological mechanisms which may underpin causal relationships between feeding frequency and energy balance (i.e. energy balance hormones). Furthermore, this approach of applying state-of-the-art analytical techniques to further progress current understanding will also provide valuable insight by examining selected health-related outcomes at a variety of levels ranging from molecular to whole-body (***Objective iii**)*. Specifically, many components and consequences of positive energy balance (e.g. poor dietary composition, sedentary behaviour and adiposity) have been well established as independent risk factors for insulin resistance and associated cardiovascular disease [56-58], with chronic low-grade inflammation strongly implicated in the latter [59]. In particular, systemic concentrations of C-reactive protein (CRP) have been shown to exhibit positive correlations with fasted glucose concentrations [60], atherosclerotic progression [61] and the incidence of initial coronary heart disease events [62]. It is therefore anticipated that the range of measures described below will address the stated objectives both from a basic and an applied science perspective to establish causal mechanisms and relationships to clinical outcomes, thus providing detailed yet practically valuable understanding in relation to public health policy and clinical practice.

### Trial Design

**Figure 2:**
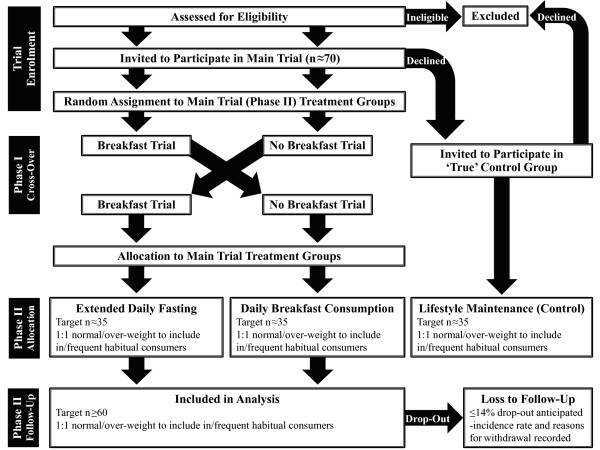
**Flow diagram illustrating progress through each phase of the trial [adapted from current CONSORT guidelines; **[63]**]**.

***Objective i ***will be addressed by way of an acute randomised cross-over comparison while ***Objective ii ***and ***Objective iii ***will be addressed via a chronic independent comparison of two parallel and randomly assigned groups, both involving a contrast between daily breakfast consumption and extended fasting. Figure 2 illustrates the course of progress through this trial consistent with current CONSORT guidelines for reporting randomised trials [63].

### Participants/Eligibility

A total cohort of 60-70 men and women will take part in the main part of the experiment, half of whom will be broadly classified as normal weight (BMI ~20-25 kg·m^-2^), whereas the other half will be obese (BMI ≥30 kg·m^-2^). This broad classification according to BMI is intended to generate two separate and diverse overall populations for subsequent more accurate and gender specific stratification based upon DEXA-derived fat-mass index (♂ FMI ≤7.5 kg·m^-2^<; ♀ FMI ≤11 kg·m^-2^<), thus controlling for differences in lean tissue [64] to allow separate analyses according to whether a given individual is ordinarily able to attain long-term energy balance. Furthermore, baseline breakfast habits will be included as a co-variate in our analysis to consider the distinct responses of frequent *versus *infrequent breakfast consumers, with frequent breakfast consumption defined as the ingestion of ≥209 kJ within two hours of waking on most days of the week. Both the above factors will be included in a stratified randomisation scheme, with generation of 4 separate block randomisation schedules to ensure an equal distribution of normal/overweight and in/frequent breakfast consumers between treatment groups. These randomisation schedules will be produced by the principal investigator (JB) using a computer-based random number generator, with full details of the overall randomisation scheme only to be published in full once group allocation is complete to complicate deciphering of the allocation sequence by those involved in trial enrolment [65]. Further procedures to conceal the allocation sequence are that the two individuals responsible for trial enrolment (JR & EC) will independently request group assignments from the principal investigator via email immediately upon verification of eligibility according to pre-stated inclusion criteria. Combining these separate requests into the same set of randomisation schedules in order of receipt therefore renders it impossible for either individual to confidently predict upcoming group assignments without prior knowledge of the other's requests. Moreover, even with this knowledge, randomisation at the point of objectively establishing eligibility ensures that the sequence in which requests are received is determined before adequate information becomes available to predict the likely responsiveness of any given individual to each treatment.

Lastly, any volunteer fulfilling all eligibility criteria (thus equivalent to the main study population) but unable to commit to the main study for other reasons (e.g. impossible to schedule trials in the required time-frame, unwilling to provide tissue samples, *etc*.) will be invited to participate as part of a 'true' control group. This group will complete only the free-living element of the study (Phase II; see later) while simply maintaining their usual lifestyle, therefore providing some background context regarding the extent to which the free-living measures themselves (i.e. dietary & physical activity monitoring) may impact outcomes but without necessitating any extended visits to the laboratory for tissue samples. These volunteers for the proposed work will be recruited from the local community (South West UK) via public advertisement (e.g. local press/radio) and will not be provided any financial incentive for participation. The protocol described herein was reviewed and approved by the National Health Service South West 3 Research Ethics Committee (10/H0106/13).

#### Inclusion Criteria

• Aged 21-60

• Body Mass Index 20-25 kg·m^-2 ^or >30 kg·m^-2^

• Able and willing to safely comply with all study procedures

• Able to provide written informed consent for participation

• Females must maintain a record of regular menstrual cycle phase or contraceptive use

• No anticipated changes in diet and/or physical activity habits during the study period (e.g. pre-planned holidays, diets/exercise plan, *etc*.)

#### Exclusion Criteria

• Any reported condition or behaviour deemed either to pose undue personal risk to the participant or introduce bias into the experiment

• Any reported use of substances which may pose undue personal risk to the participants or introduce bias into the experiment

• Any individual whose habitual lifestyle does not conform to a standard sleep-wake cycle (e.g. shift workers)

• Simultaneous or recent (i.e. last 3 months) participation in another clinical trial or blood donation, to allow full recovery of blood volume [66]

• Any reported recent (i.e. last 6 months) shift (>1 kg) in body mass

• Any reported tendency towards keloid scarring

• Any reported bleeding disorder

• Females known to be pregnant or planning to become so over the course of the study

• Females with oral or implanted contraceptives fitted within 6 months of participation [67]

• Females who are breastfeeding

### Statistical Approach/Power Estimation

The number of research participants to be recruited was estimated based upon a worthwhile effect dictated by the smallest shift in energy balance necessary to induce chronic weight loss. Specifically, one of the most similar studies to the proposed work applied doubly-labelled water to measure average daily energy expenditure in a population not dissimilar to that planned [38], from which a mean of ~12000 kJ·d^-1 ^might be expected with a standard deviation in the region of ~2000 kJ·d^-1^. The daily breakfast to be consumed in this trial will provide in excess of ~3000 kJ·d^-1 ^and our pilot work using this breakfast indicates that this is likely to stimulate metabolism in the lead up to lunch by ~150 kJ·d^-1 ^and reduce energy intake at lunch by ~150 kJ·d^-1^, relative to continued fasting. Therefore, for breakfast consumption to exert a worthwhile effect on net energy balance (i.e. sufficient to compensate for the energy it provides directly), physical activity energy expenditure would need to increase and/or subsequent energy intake to decrease by a further 2700 kJ·d^-1^. Using the above figures, a worthwhile increase in energy expenditure would require ~14 participants in each treatment group to confer a 90% probability of detecting such an effect statistically using a two-tailed *t*-test with an alpha level of 0.05. A total of 70 participants will therefore be recruited for the main study treatment arms in view of the 14% drop-out rate reported by Schlundt *et al*. (1992) when placing similar demands on volunteers [51], thus aiming to achieve at least 60 participants to provide sufficient statistical power even when the overall breakfast *versus *no breakfast groups are stratified according to FMI (with baseline breakfast habits, gender and age included as co-variates in the analysis). Nonetheless, to minimise the negative impact of loss to follow-up on validity, participants will be advised at trial enrolment to very carefully consider the required investment of time and effort relative to their current and forthcoming commitments outside of the trial, such that exclusions based on likely withdrawal can be made prior to randomisation [68]. Any unforeseen circumstances or withdrawal of consent subsequently resulting in loss to follow-up will be documented fully in the final trial report. Data collected from these individuals to the point of loss will then be compared with those who complete each treatment to determine whether the overall conclusions of the trial may be generalised beyond populations reflective of the latter (i.e. to anyone willing to be randomised).

As with the primary outcome measure, secondary variables will also be analysed using either paired or independent *t*-tests applied to simple summary statistics between groups or trials, respectively (e.g. fasted/peak values, incremental area under curve, average value over 24 h, *etc*.). Given that these sample size estimations are based on a primary outcome that inherently displays a relatively large inter-individual variability, it is anticipated that the more consistent responses of secondary variables will also be detectable with the selected sample size.

### Experimental Protocol

***Objective i - ***The first phase of the proposed work (Phase I) will involve two laboratory-based examinations of the acute metabolic and behavioural responses to extended fasting relative to ingestion of a standardised breakfast. These trials will be applied in a randomised and counterbalanced order with 3-28 days interval except for eumenorrheic women, whose trials will be separated by 28 ± 2 days and only at least 3 and at most 10 days after the onset of menses (i.e. follicular phase) to ensure that the effects of menstrual cycle on the majority of hormones and therefore resting metabolic rate (RMR) and appetite will be both minimal and standardised between trials [69-71]. These acute trials will be conducted in our resting metabolism laboratory in accordance with current guidelines for best practice in measuring resting metabolic rate [72]. Most notably, ambient temperature in this laboratory is maintained between 20 and 25°C (with intra-individual trials standardised within 2°C) and participants will arrive for testing at 8 am (±1 h) in a 10 hour fasted state but having ingested 1 pint of plain water upon waking following standardised physical activity and diet (the latter to incorporate either daily breakfast or extended fasting according to the group randomisation described later, thus permitting meaningful comparisons with follow-up visits). Repeated 5 minute resting expired gas samples will be collected over 30 minutes (after 20 minutes of quiet rest) to establish RMR and substrate selection before a cannula will be fitted to an antecubital vein for the acquisition of a baseline 15 ml blood sample, along with further samples throughout that day.

At this stage, participants will provide ratings of hunger and appetite using validated visual analogue scales [73], for subsequent follow-up post-breakfast and also pre-post lunch and at the end of day. They will then receive either no breakfast or a typical standardised breakfast (composed of cereal, toast and orange juice) with the opposite treatment applied in each participant's second trial). The specific food and macronutrient composition of this breakfast has been described previously [74] and provides quantities intended to deliver 14 mg carbohydrate per kJ of each individual's previously established RMR. Fifteen minutes later or upon completion of the breakfast (if longer than 15 minutes is required) a 3 hour post-prandial period will commence, involving collection of all urine output (for determination of urea nitrogen excretion) with hourly expired 5 minute gas samples to assess changes in substrate metabolism (i.e. carbohydrate, lipid and protein oxidation) and DIT (i.e. post-prandial energy expenditure minus RMR). Fifteen millilitre blood samples (including 5 ml waste) will also be drawn 15, 30 & 60 minutes post-breakfast, then at hourly intervals to determine systemic concentrations of: glucose; lactate; insulin; NEFA; urea; 'energy balance hormones' that are known to play a central role in the regulation of metabolic rate, appetite and spontaneous physical activity (e.g. free thyroxine, adiponectin, CCK, total/acylated ghrelin, leptin & PYY); and also cytokine concentrations (e.g. interleukin-6 and TNF-α), in view of recently documented differences in post-prandial inflammatory responses to varied meals across different populations [75] (with low/high density lipoprotein cholesterol and CRP only followed-up at the 3 hour time-point). Participants will then be provided with a test meal of standardised composition (1 kg cooked Sainsbury's™ penne pasta and Ragu™ traditional tomato sauce; prepared at a ratio of 1:1 uncooked mass) along with *ad libitum *plain water (although participants will ingest the same volume of water as in their first trial during all subsequent visits), which we have previously employed during pilot work to estimate likely differences in voluntary energy intake. At this stage, participants will be left alone in the laboratory and receive a recorded message stating: "We ask that you continue eating until you have satisfied your hunger. The lunch will remain in front of your for at least 30 minutes, at which point the post-lunch timer will be started, although you will be allowed to continue eating if you are still hungry."

The bowl of pasta will be refilled every 10 minutes to minimise any visual feedback in the regulation of appetite. To gain additional insight regarding psychological reward and food hedonics, a validated labelled magnitude scale [76] will be administered following the first and last mouthful of this meal to reliably assess the degree of 'pleasantness' associated with ingestive behaviour. The 3 hour post-prandial period will then be repeated exactly as following breakfast (but without the 15-30 minute samples) to track metabolic responses to lunch.

***Objective ii - ***Phase II of testing will commence within 3-28 days after completion of each participants' second trial under Phase I of testing (thus using participants' breakfast trial along with the measures described above as a baseline). This trial schedule will again be used for all participants other than for eumenorrheic women, who will follow the same restrictions as described above and therefore occasionally be required to complete their first trial under Phase II prior to the completing the second trial under Phase I. In such cases, these participants' 6-week intervention will simply be deferred for two weeks (i.e. enabling completion of Phase I) to allow follow-up measures to be taken at the same stage in the menstrual cycle. The first visit under Phase II will begin with participants again arriving in the laboratory at 8 am (±1 h) having adhered to the same standardisation procedures as described for Phase I. Measurements of body mass and adiposity based on hip and waist circumference (i.e. widest gluteal girth & mid-point between lowest rib and iliac crest, respectively), sagittal abdominal height (using a Holtain-Kahn calliper at the iliac crest) and body composition via DEXA (Hologic Discovery W) will also be made at this visit, before a small (~1 g) sample of subcutaneous adipose tissue will be acquired using a 14 G needle to determine basal expression of key genes related to appetite and physical activity regulation (e.g. adiponectin and leptin) and energy expenditure (e.g. UCPs).

This second phase of data collection will involve each participant being randomly assigned either to an extended daily fasting group (only plain water permitted until 1200 each day) or a breakfast consumption group (prescribed intake of ≥3000 kJ before 1100 each day, to include at least 1500 kJ within two hours of waking) for a period of 6 weeks [a duration previously shown to be sufficient for dietary modification to induce detectable changes in energy balance and body composition; [35]. To facilitate this process, participants assigned to the breakfast group will be provided with detailed examples of appropriately energetic breakfasts and the energy content of other typical breakfast foods, although the breakfasts consumed will ultimately be self-selected by each individual on a daily basis. Upon completion of this 6 week dietary modification, participants will return to the laboratory for follow-up of all anthropometric measures and a second subcutaneous adipose tissue biopsy to determine whether the intervention has altered the expression of those genes measured at baseline (described above).

In relation to the stated objectives, this design therefore examines whether and how any acute alterations in energy balance can culminate in chronic changes in energy balance (i.e. the acute effects of each treatment may be modified as participants become accustomed and adapt to it). To further inform such questions, during the first and last week of the intervention participants will maintain detailed weighed records of their habitual food and fluid intakes for subsequent analysis of daily energy and macro-nutrient intakes using dietary analysis software (CompEat Pro). Additional data regarding feeding patterns will be gathered via analysis of time of day, daily frequency and energy content of individual eating occasions, with meals defined as ingestion of ≥1256 kJ at any given eating occasion and snacks defined as ingestion of <1256 kJ more than 45 minutes before or after a meal [77]. All participants will receive telephone reminders to ensure appropriate compliance to the dietary record process, as has been advocated and applied to good effect by others [24]. The food diaries provided to participants will also be accompanied by the same validated labelled magnitude scale [76] that participants will have used during the first phase of testing to provide a rating of 'pleasantness' following the first and last mouthful of their lunchtime meal, thus informing research questions regarding psychological reward and food hedonics. Concurrent to these two periods of dietary recording, participants will also be fitted with a combined heart-rate/accelerometer (Actiheart, CamNtech) in order to accurately record energy expenditure/physical activity habits [54,55] for the entire duration of each 7 day assessment period (which will also be complemented by daily records of participants physical in/activities and sleep/waking times). Importantly, at the point when this physical activity monitor is fitted, participants will be provided the following message both verbally and in writing to ensure that only genuinely meaningful behavioural responses are recorded: "Your lifestyle choices during this free-living monitoring period are central to this study. We are interested in any natural changes in your diet and/or physical activity habits, which you may or may not make in response to the intervention. This monitoring period has been carefully scheduled to avoid any pre-planned changes in these habits, such as a holiday or diet/exercise plan. You should inform us immediately if unforeseen factors external to the study may influence your lifestyle."

After each participant's follow-up trial under Phase II, participants will continue adhering to their assigned intervention for at least 2 days to allow for the 48 hour dietary control (although up to 7 days will be permitted in participants for whom either the menstrual cycle need not be controlled for or when both trials can still occur within the follicular phase) before returning to the laboratory to repeat their breakfast trial exactly as described in relation to Objective i. This trial will therefore inform whether acute metabolic and/or behavioural effects may be modified following chronic exposure to each treatment. A formal exit interview will also be incorporated into this final visit during the afternoon post-prandial period to obtain qualitative data regarding participants' experiences of the study and motives for any perceived changes in behaviour.

***Objective iii - ***In relation to this objective, relevant data will be gathered during the second phase of testing to explore how interactions between extended daily fasting may relate to insulin resistance and cardiovascular disease risk (whether related to or independent of changes in energy balance). From a whole-body perspective, highly relevant data will be gathered during the first and last week of the 6 week intervention using a subcutaneous continuous glucose monitor (iPro, Medtronic) to record 24 hour glucose profiles for each participant (thus revealing whether either treatment alters either the average daily glucose concentration or the magnitude of hyperglycaemic excursions following meals). This real-world indication of glycaemic control will be complimented not only by simple comparison of changes in fasted glucose/insulin concentrations changes over the 6 weeks but also by way of an oral glucose tolerance test (OGTT) that will be conducted at baseline and follow-up in Phase II, immediately following each adipose tissue sample. At a more reductionist level, the adipose tissue samples will be subjected to further analysis to determine the sensitivity of this particular tissue to insulin. Specifically, on the day that these samples are collected, adipocytes will be isolated by collagenase digestion before determination of [U-^14^C]-D-glucose uptake at basal, submaximal (50 pmol•l^-1^) and maximal (20 nmol•l^-1^) insulin concentrations. This glucose uptake assay has been shown to accurately reflect 3-O-methylglucose transport under the conditions described [78,79] and the resultant data will be expressed both as pmol•min^-1 ^relative both to lipid mass [80] and, following analysis of cell size, to cell surface area. This will be in addition to subsequent analyses for the total protein content of GLUT4 and Akt in this tissue and the expression of key genes implicated in lipolysis (e.g. HSL and ATGL), lipogenesis (e.g. PPARγ, PGC1a, SREBP1c) and more generally in glucose uptake and oxidation (e.g. GLUT4, PDK4, AMPKα-1/2, resistin, RBP4, leptin and adiponectin) and/or insulin signalling (e.g. IRS1/2, PI3K, Akt and TBC1 domain family member 4), thus informing whether there is any adaptive response to the intervention at the level of adipose tissue gene expression and protein content.

Finally, in relation to cardiovascular disease risk, measurements of blood lipid profiles, cytokine responses, CRP and blood pressure (DINAMAP Pro 100-400 V2, UK) will be contrasted between participants' baseline and final follow-up trials, as will the expression of other relevant genes in adipose tissue samples (e.g. interleukin-6 and TNF-α). All hormones/cytokines to be measured in the proposed work will be quantified via ELISA, with whole blood glucose determined using a YSI analyser, plasma glucose, NEFA, cholesterol and urea measured using a spectrophotometer and gene expression using TaqMan^® ^Real-Time PCR.

## Discussion

Despite clear support for a positive relationship between extended daily fasting, energy balance and a range of inter-related negative health consequences, there remains a distinct lack of evidence regarding any direct causal relationships or mechanisms of action. The novel data generated by this study will be critical both to the progression of scientific understanding and to the application of these findings within a public health setting. In particular, very simple and easily adopted public health messages could be communicated based on the findings of this study, not only in terms of whether breakfast consumption/omission represents an effective strategy for weight loss or improved health in different populations but also the mechanisms identified can be facilitated to maximise such benefits.

## Abbreviations

Akt: also known as Protein Kinase B (PKB); AMPK: Adenosine Monophosphate-Activated Protein Kinase; ATGL: Adipose Triglyceride Lipase; BMI: Body Mass Index; CCK: Cholecystokinin; CRP: C-Reactive Protein; DEXA: Dual Energy X-ray Absorptiometry; DIT: Diet induced Thermogenesis; also known as the Thermic Effect of Feeding (TEF); ELISA: Enzyme Linked Immuno-Sorbent Assays; FMI: Fat Mass Index; GLUT: Glucose Transporter; HSL: Hormone Sensitive Lipase; IRS: Insulin Receptor Substrate; NEFA: Non-Esterified Fatty Acids; OGTT: Oral Glucose Tolerance Test; PCR: Polymerase Chain Reaction; PDK: Pyruvate Dehydrogenase Kinase; PGC: Peroxisome Proliferator-Activated Receptor Coactivator; PI3K: Phosphatidylinositol 3-kinase; PPAR: Peroxisome Proliferator-Activated Receptor; PYY: Peptide YY; RBP4: Retinol Binding Protein 4; RMR: Resting Metabolic Rate; SREBP: Sterol Regulatory Element-Binding Protein; TBC: Tre-2, Bub2p and Cdc16p; TNF-α: Tumor Necrosis Factor-α; UCP: Uncoupling Protein.

## Declaration of Competing interests

The authors declare that they have no competing interests.

## Authors' contributions

JB: principal investigator - conceived of the research question and drafted this manuscript. DT: project co-ordinator - provided advice and informed revisions to protocol. JR: informed revisions to protocol while managing the project/collecting data on a daily basis. EC: informed revisions to protocol while managing the project/collecting data on a daily basis. MJ: informed dietary analysis strategy while analysing diets and collecting data on a daily basis. GH: advisor and collaborator in analysis of adipocyte insulin sensitivity and metabolism. KT: advisor and collaborator in analysis of adipocyte insulin sensitivity and metabolism. All authors contributed to revisions of this manuscript and approved the final version.

## References

[B1] Fabry P Hejl Z Fodor J Braun T Zvolankova K The frequency of meals. Its relation to overweight, hypercholesterolaemia, and decreased glucose-tolerance Lancet 1964 2 614 615 14186149 10.1016/s0140-6736(64)90510-0

[B2] Siega-Riz AM Popkin BM Carson T Trends in breakfast consumption for children in the United States from 1965-1991 Am J Clin Nutr 1998 67 748S 756S 9537624 10.1093/ajcn/67.4.748S

[B3] Ruxton CH Kirk TR Breakfast: a review of associations with measures of dietary intake, physiology and biochemistry Br J Nutr 1997 78 199 213 10.1079/BJN19970140 9301411

[B4] de la Hunty A Ashwell M Are people who regularly eat breakfast cereals slimmer than those who don't? A systematic review of the evidence Nutrition Bulletin 2007 32 118 128 10.1111/j.1467-3010.2007.00638.x

[B5] Gibson S Micronutrient intakes, micronutrient status and lipid profiles among young people consuming different amounts of breakfast cereals: further analysis of data from the National Diet and Nutrition Survey of Young People aged 4 to 18 years Public Health Nutrition 2003 6 815 820 14641953 10.1079/phn2003493

[B6] Duval K Strychar I Cyr MJ Prud'homme D Rabasa-Lhoret R Doucet E Physical activity is a confounding factor of the relation between eating frequency and body composition Am J Clin Nutr 2008 88 1200 1205 18996853 10.3945/ajcn.2008.26220

[B7] Deshmukh-Taskar PR Nicklas TA O'Neil CE Keast DR Radcliffe JD Cho S The relationship of breakfast skipping and type of breakfast consumption with nutrient intake and weight status in children and adolescents: the National Health and Nutrition Examination Survey 1999-2006 J Am Diet Assoc 2010 110 869 878 10.1016/j.jada.2010.03.023 20497776

[B8] Matthys C De Henauw S Bellemans M De Maeyer M De Backer G Breakfast habits affect overall nutrient profiles in adolescents Public Health Nutr 2007 10 413 421 17362538 10.1017/S1368980007248049

[B9] Utter J Scragg R Mhurchu CN Schaaf D At-home breakfast consumption among New Zealand children: associations with body mass index and related nutrition behaviors J Am Diet Assoc 2007 107 570 576 10.1016/j.jada.2007.01.010 17383261

[B10] Keski-Rahkonen A Kaprio J Rissanen A Virkkunen M Rose RJ Breakfast skipping and health-compromising behaviors in adolescents and adults Eur J Clin Nutr 2003 57 842 853 10.1038/sj.ejcn.1601618 12821884

[B11] Timlin MT Pereira MA Story M Neumark-Sztainer D Breakfast eating and weight change in a 5-year prospective analysis of adolescents: Project EAT (Eating Among Teens) Pediatrics 2008 121 e638 645 10.1542/peds.2007-1035 18310183

[B12] Albertson AM Anderson GH Crockett SJ Goebel MT Ready-to-eat cereal consumption: its relationship with BMI and nutrient intake of children aged 4 to 12 years J Am Diet Assoc 2003 103 1613 1619 10.1016/j.jada.2003.09.020 14647087

[B13] Bertrais S Polo Luque ML Preziosi P Fieux B Torra De Flot M Galan P Hercberg S Contribution of ready-to-eat cereals to nutrition intakes in French adults and relations with corpulence Ann Nutr Metab 2000 44 249 255 10.1159/000046692 11146332

[B14] Cho S Dietrich M Brown CJ Clark CA Block G The effect of breakfast type on total daily energy intake and body mass index: results from the Third National Health and Nutrition Examination Survey (NHANES III) J Am Coll Nutr 2003 22 296 302 12897044 10.1080/07315724.2003.10719307

[B15] Gibson SA O'Sullivan KR Breakfast cereal consumption patterns and nutrient intakes of British schoolchildren J R Soc Health 1995 115 366 370 10.1177/146642409511500608 8568785

[B16] Ortega RM Requejo AM Lopez-Sobaler AM Quintas ME Andres P Redondo MR Navia B Lopez-Bonilla MD Rivas T Difference in the breakfast habits of overweight/obese and normal weight schoolchildren Int J Vitam Nutr Res 1998 68 125 132 9565828

[B17] Song WO Chun OK Obayashi S Cho S Chung CE Is consumption of breakfast associated with body mass index in US adults? J Am Diet Assoc 2005 105 1373 1382 10.1016/j.jada.2005.06.002 16129078

[B18] Barton BA Eldridge AL Thompson D Affenito SG Striegel-Moore RH Franko DL Albertson AM Crockett SJ The relationship of breakfast and cereal consumption to nutrient intake and body mass index: the National Heart, Lung, and Blood Institute Growth and Health Study J Am Diet Assoc 2005 105 1383 1389 10.1016/j.jada.2005.06.003 16129079

[B19] Bazzano LA Song Y Bubes V Good CK Manson JE Liu S Dietary intake of whole and refined grain breakfast cereals and weight gain in men Obes Res 2005 13 1952 1960 10.1038/oby.2005.240 16339127

[B20] Berkey CS Rockett HR Gillman MW Field AE Colditz GA Longitudinal study of skipping breakfast and weight change in adolescents Int J Obes Relat Metab Disord 2003 27 1258 1266 10.1038/sj.ijo.0802402 14513075

[B21] Kant AK Schatzkin A Graubard BI Ballard-Barbash R Frequency of eating occasions and weight change in the NHANES I Epidemiologic Follow-up Study Int J Obes Relat Metab Disord 1995 19 468 474 8520636

[B22] Little P Barnett J Margetts B Kinmonth AL Gabbay J Thompson R Warm D Warwick H Wooton S The validity of dietary assessment in general practice J Epidemiol Community Health 1999 53 165 172 10.1136/jech.53.3.165 10396494 PMC1756848

[B23] Summerbell CD Moody RC Shanks J Stock MJ Geissler C Relationship between feeding pattern and body mass index in 220 free-living people in four age groups Eur J Clin Nutr 1996 50 513 519 8863011

[B24] Dreon DM Frey-Hewitt B Ellsworth N Williams PT Terry RB Wood PD Dietary fat:carbohydrate ratio and obesity in middle-aged men Am J Clin Nutr 1988 47 995 1000 3376914 10.1093/ajcn/47.6.995

[B25] Turconi G Bazzano R Caramella R Porrini M Crovetti R Lanzola E The effects of high intakes of fibre ingested at breakfast on satiety Eur J Clin Nutr 1995 49 281 285 8549549

[B26] Farshchi HR Taylor MA Macdonald IA Deleterious effects of omitting breakfast on insulin sensitivity and fasting lipid profiles in healthy lean women Am J Clin Nutr 2005 81 388 396 15699226 10.1093/ajcn.81.2.388

[B27] Smeets AJ Westerterp-Plantenga MS Acute effects on metabolism and appetite profile of one meal difference in the lower range of meal frequency Br J Nutr 2008 99 1316 1321 18053311 10.1017/S0007114507877646

[B28] Speechly DP Buffenstein R Greater appetite control associated with an increased frequency of eating in lean males Appetite 1999 33 285 297 10.1006/appe.1999.0265 10625522

[B29] Wyatt HR Grunwald GK Mosca CL Klem ML Wing RR Hill JO Long-term weight loss and breakfast in subjects in the National Weight Control Registry Obes Res 2002 10 78 82 10.1038/oby.2002.13 11836452

[B30] Smith KJ Gall SL McNaughton SA Blizzard L Dwyer T Venn AJ Skipping breakfast: longitudinal associations with cardiometabolic risk factors in the Childhood Determinants of Adult Health Study Am J Clin Nutr 2010 92 1316 1325 10.3945/ajcn.2010.30101 20926520

[B31] Dallosso HM Murgatroyd PR James WP Feeding frequency and energy balance in adult males Human Nutrition:Clinical Nutrition 1982 36C 25 39 7076516

[B32] Farshchi HR Taylor MA Macdonald IA Decreased thermic effect of food after an irregular compared with a regular meal pattern in healthy lean women Int J Obes Relat Metab Disord 2004 28 653 660 10.1038/sj.ijo.0802616 15085170

[B33] Garrow JS Durrant M Blaza S Wilkins D Royston P Sunkin S The effect of meal frequency and protein concentration on the composition of the weight lost by obese subjects Br J Nutr 1981 45 5 15 10.1079/BJN19810072 7470437

[B34] Hubert P King NA Blundell JE Uncoupling the effects of energy expenditure and energy intake: appetite response to short-term energy deficit induced by meal omission and physical activity Appetite 1998 31 9 19 10.1006/appe.1997.0148 9716432

[B35] Keim NL Van Loan MD Horn WF Barbieri TF Mayclin PL Weight loss is greater with consumption of large morning meals and fat-free mass is preserved with large evening meals in women on a controlled weight reduction regimen J Nutr 1997 127 75 82 9040548 10.1093/jn/127.1.75

[B36] Martin A Normand S Sothier M Peyrat J Louche-Pelissier C Laville M Is advice for breakfast consumption justified? Results from a short-term dietary and metabolic experiment in young healthy men Br J Nutr 2000 84 337 344 10.1017/S0007114500001616 10967612

[B37] LeBlanc J Mercier I Nadeau A Components of postprandial thermogenesis in relation to meal frequency in humans Can J Physiol Pharmacol 1993 71 879 883 10.1139/y93-133 8180882

[B38] Verboeket-van de Venne WP Westerterp KR Kester AD Effect of the pattern of food intake on human energy metabolism Br J Nutr 1993 70 103 115 10.1079/BJN19930108 8399092

[B39] Tai MM Castillo P Pi-Sunyer FX Meal size and frequency: effect on the thermic effect of food Am J Clin Nutr 1991 54 783 787 1951147 10.1093/ajcn/54.5.783

[B40] Belko A Barbieri T Effect of meal size and frequency on the thermic effect of food Nutrition Research 1987 7 237 242 10.1016/S0271-5317(87)80013-1

[B41] Kinabo JL Durnin JV Effect of meal frequency on the thermic effect of food in women Eur J Clin Nutr 1990 44 389 395 2387273

[B42] Taylor MA Garrow JS Compared with nibbling, neither gorging nor a morning fast affect short-term energy balance in obese patients in a chamber calorimeter Int J Obes Relat Metab Disord 2001 25 519 528 10.1038/sj.ijo.0801572 11319656

[B43] Arnold LM Ball MJ Duncan AW Mann J Effect of isoenergetic intake of three or nine meals on plasma lipoproteins and glucose metabolism Am J Clin Nutr 1993 57 446 451 8438781 10.1093/ajcn/57.3.446

[B44] Farshchi HR Taylor MA Macdonald IA Regular meal frequency creates more appropriate insulin sensitivity and lipid profiles compared with irregular meal frequency in healthy lean women Eur J Clin Nutr 2004 58 1071 1077 10.1038/sj.ejcn.1601935 15220950

[B45] Schusdziarra V Hausmann M Wittke C Mittermeier J Kellner M Naumann A Wagenpfeil S Erdmann J Impact of breakfast on daily energy intake - an analysis of absolute versus relative breakfast calories Nutrition Journal 2011 10 5 10.1186/1475-2891-10-5 21241465 PMC3034667

[B46] Stote KS Baer DJ Spears K Paul DR Harris GK Rumpler WV Strycula P Najjar SS Ferrucci L Ingram DK A controlled trial of reduced meal frequency without caloric restriction in healthy, normal-weight, middle-aged adults Am J Clin Nutr 2007 85 981 988 17413096 10.1093/ajcn/85.4.981 PMC2645638

[B47] Chapelot D Marmonier C Aubert R Allegre C Gausseres N Fantino M Louis-Sylvestre J Consequence of omitting or adding a meal in man on body composition, food intake, and metabolism Obesity 2006 14 215 227 10.1038/oby.2006.28 16571846

[B48] Verboeket-van de Venne WP Westerterp KR Frequency of feeding, weight reduction and energy metabolism Int J Obes Relat Metab Disord 1993 17 31 36 8383639

[B49] Halberg N Henriksen M Soderhamn N Stallknecht B Ploug T Schjerling P Dela F Effect of intermittent fasting and refeeding on insulin action in healthy men J Appl Physiol 2005 99 2128 2136 10.1152/japplphysiol.00683.2005 16051710

[B50] Soeters MR Lammers NM Dubbelhuis PF Ackermans M Jonkers-Schuitema CF Fliers E Sauerwein HP Aerts JM Serlie MJ Intermittent fasting does not affect whole-body glucose, lipid, or protein metabolism Am J Clin Nutr 2009 90 1244 1251 10.3945/ajcn.2008.27327 19776143

[B51] Schlundt DG Hill JO Sbrocco T Pope-Cordle J Sharp T The role of breakfast in the treatment of obesity: a randomized clinical trial Am J Clin Nutr 1992 55 645 651 1550038 10.1093/ajcn/55.3.645

[B52] Carlson O Martin B Stote KS Golden E Maudsley S Najjar SS Ferrucci L Ingram DK Longo DL Rumpler WV Impact of reduced meal frequency without caloric restriction on glucose regulation in healthy, normal-weight middle-aged men and women Metabolism 2007 56 1729 1734 10.1016/j.metabol.2007.07.018 17998028 PMC2121099

[B53] Summerbell CD Moody RC Shanks J Stock MJ Geissler C Sources of energy from meals versus snacks in 220 people in four age groups Eur J Clin Nutr 1995 49 33 41 7713049

[B54] Corder K Brage S Mattocks C Ness A Riddoch C Wareham NJ Ekelund U Comparison of two methods to assess PAEE during six activities in children Med Sci Sports Exerc 2007 39 2180 2188 10.1249/mss.0b013e318150dff8 18046189

[B55] Thompson D Batterham AM Bock S Robson C Stokes K Assessment of low-to-moderate intensity physical activity thermogenesis in young adults using synchronized heart rate and accelerometry with branched-equation modeling J Nutr 2006 136 1037 1042 16549471 10.1093/jn/136.4.1037

[B56] Weinstein AR Sesso HD Lee IM Rexrode KM Cook NR Manson JE Buring JE Gaziano JM The joint effects of physical activity and body mass index on coronary heart disease risk in women Arch Intern Med 2008 168 884 890 10.1001/archinte.168.8.884 18443265

[B57] Ceriello A Impaired glucose tolerance and cardiovascular disease: the possible role of post-prandial hyperglycemia Am Heart J 2004 147 803 807 10.1016/j.ahj.2003.11.020 15131534

[B58] Iqbal R Anand S Ounpuu S Islam S Zhang X Rangarajan S Chifamba J Al-Hinai A Keltai M Yusuf S Dietary patterns and the risk of acute myocardial infarction in 52 countries: results of the INTERHEART study Circulation 2008 118 1929 1937 10.1161/CIRCULATIONAHA.107.738716 18936332

[B59] Pearson TA Mensah GA Alexander RW Anderson JL Cannon RO Criqui M Fadl YY Fortmann SP Hong Y Myers GL Markers of inflammation and cardiovascular disease: application to clinical and public health practice Circulation 2003 107 499 511 3rd 10.1161/01.CIR.0000052939.59093.45 12551878

[B60] Aronson D Bartha P Zinder O Kerner A Shitman E Markiewicz W Brook GJ Levy Y Association between fasting glucose and C-reactive protein in middle-aged subjects Diabet Med 2004 21 39 44 10.1046/j.1464-5491.2003.01084.x 14706052

[B61] Kang ES Kim HJ Kim YM Lee S Cha BS Lim SK Lee HC Serum high sensitivity C-reactive protein is associated with carotid intima-media thickness in type 2 diabetes Diabetes Res Clin Pract 2004 66 S115 120 15563960 10.1016/j.diabres.2004.05.009

[B62] Rutter MK Meigs JB Sullivan LM D'Agostino RB Sr Wilson PW C-reactive protein, the metabolic syndrome, and prediction of cardiovascular events in the Framingham Offspring Study Circulation 2004 110 380 385 10.1161/01.CIR.0000136581.59584.0E 15262834

[B63] Schulz KF Altman DG Moher D CONSORT 2010 Statement: updated guidelines for reporting parallel group randomised trials Trials 2010 11 32 10.1186/1745-6215-11-32 21350618 PMC3043330

[B64] Kelly TL Wilson KE Heymsfield SB Dual energy X-Ray absorptiometry body composition reference values from NHANES PLoS ONE 2009 4 e7038 10.1371/journal.pone.0007038 19753111 PMC2737140

[B65] Schulz KF Grimes DA Unequal group sizes in randomised trials: guarding against guessing Lancet 2002 359 966 970 10.1016/S0140-6736(02)08029-7 11918933

[B66] Pottgiesser T Specker W Umhau M Dickhuth HH Roecker K Schumacher YO Recovery of hemoglobin mass after blood donation Transfusion (Paris) 2008 48 1390 1397 10.1111/j.1537-2995.2008.01719.x 18466177

[B67] Biswas A Viegas OA Coeling HJ Bennink Korver T Ratnam SS Implanon contraceptive implants: effects on carbohydrate metabolism Contraception 2001 63 137 141 10.1016/S0010-7824(01)00182-2 11368985

[B68] Schulz KF Grimes DA Sample size slippages in randomised trials: exclusions and the lost and wayward Lancet 2002 359 781 785 10.1016/S0140-6736(02)07882-0 11888606

[B69] Buffenstein R Poppitt SD McDevitt RM Prentice AM Food intake and the menstrual cycle: a retrospective analysis, with implications for appetite research Physiol Behav 1995 58 1067 1077 10.1016/0031-9384(95)02003-9 8623004

[B70] Lissner L Stevens J Levitsky DA Rasmussen KM Strupp BJ Variation in energy intake during the menstrual cycle: implications for food-intake research Am J Clin Nutr 1988 48 956 962 3421205 10.1093/ajcn/48.4.956

[B71] Solomon SJ Kurzer MS Calloway DH Menstrual cycle and basal metabolic rate in women Am J Clin Nutr 1982 36 611 616 7124662 10.1093/ajcn/36.4.611

[B72] Compher C Frankenfield D Keim N Roth-Yousey L Best practice methods to apply to measurement of resting metabolic rate in adults: a systematic review J Am Diet Assoc 2006 106 881 903 10.1016/j.jada.2006.02.009 16720129

[B73] Stubbs RJ Hughes DA Johnstone AM Rowley E Reid C Elia M Stratton R Delargy H King N Blundell JE The use of visual analogue scales to assess motivation to eat in human subjects: a review of their reliability and validity with an evaluation of new hand-held computerized systems for temporal tracking of appetite ratings Br J Nutr 2000 84 405 415 10.1017/S0007114500001719 11103211

[B74] Chryssanthopoulos C Williams C Nowitz A Bogdanis G Skeletal muscle glycogen concentration and metabolic responses following a high glycaemic carbohydrate breakfast J Sports Sci 2004 22 1065 1071 10.1080/02640410410001730007 15801500

[B75] Manning PJ Sutherland WH McGrath MM de Jong SA Walker RJ Williams MJ Postprandial cytokine concentrations and meal composition in obese and lean women Obesity 2008 16 2046 2052 10.1038/oby.2008.334 19186329

[B76] Haase L Cerf-Ducastel B Buracas G Murphy C On-line psychophysical data acquisition and event-related fMRI protocol optimized for the investigation of brain activation in response to gustatory stimuli J Neurosci Methods 2007 159 98 107 10.1016/j.jneumeth.2006.07.009 16978702

[B77] de Castro JM Accommodation of particular foods or beverages into spontaneously ingested evening meals Appetite 1994 23 57 66 10.1006/appe.1994.1034 7826057

[B78] Foley JE Kashiwagi A Verso MA Reaven G Andrews J Improvement in in vitro insulin action after one month of insulin therapy in obese noninsulin-dependent diabetics. Measurements of glucose transport and metabolism, insulin binding, and lipolysis in isolated adipocytes J Clin Invest 1983 72 1901 1909 10.1172/JCI111153 6358258 PMC437029

[B79] Kashiwagi A Verso MA Andrews J Vasquez B Reaven G Foley JE In vitro insulin resistance of human adipocytes isolated from subjects with noninsulin-dependent diabetes mellitus J Clin Invest 1983 72 1246 1254 10.1172/JCI111080 6355180 PMC370408

[B80] Liu SC Wang Q Lienhard GE Keller SR Insulin receptor substrate 3 is not essential for growth or glucose homeostasis J Biol Chem 1999 274 18093 18099 10.1074/jbc.274.25.18093 10364263

